# Validity of self-reported weight and height: a cross-sectional study among Malaysian adolescents

**DOI:** 10.1186/s12874-017-0362-0

**Published:** 2017-06-02

**Authors:** C. C. Kee, K. H. Lim, M. G. Sumarni, C. H. Teh, Y. Y. Chan, M. I. Nuur Hafizah, Y. K. Cheah, E. O. Tee, Y. Ahmad Faudzi, M. Amal Nasir

**Affiliations:** 1 Epidemiology and Biostatistics Unit, Institute for Medical Research, Jalan Pahang, 50588 Kuala Lumpur, Malaysia; 2 Institute for Public Health, Jalan Bangsar, Federal Hill, 50590 Kuala Lumpur, Malaysia; 3 Universiti Utara Malaysia, UUM, 06010 Sintok, Kedah Malaysia; 4 Allied Health Sciences College Sg. Buloh, Jalan Hospital, 47000 Sg. Buloh, Selangor Malaysia

**Keywords:** Validity, Self-report, Weight, Height, Body mass index, Adolescents

## Abstract

**Background:**

Self-reported weight and height are commonly used in lieu of direct measurements of weight and height in large epidemiological surveys due to inevitable constraints such as budget and human resource. However, the validity of self-reported weight and height, particularly among adolescents, needs to be verified as misreporting could lead to misclassification of body mass index and therefore overestimation or underestimation of the burden of BMI-related diseases. The objective of this study was to determine the validity of self-reported weight and height among Malaysian secondary school children.

**Methods:**

Both self-reported and directly measured weight and height of a subgroup of 663 apparently healthy schoolchildren from the Malaysian Adolescent Health Risk Behaviour (MyAHRB) survey 2013/2014 were analysed. Respondents were required to report their current body weight and height via a self-administrative questionnaire before they were measured by investigators. The validity of self-reported against directly measured weight and height was examined using intraclass correlation coefficient (ICC), the Bland-Altman plot and weighted Kappa statistics.

**Results:**

There was very good intraclass correlation between self-reported and directly measured weight [*r* = 0.96, 95% confidence interval (CI): 0.93, 0.97] and height (*r* = 0.94, 95% CI: 0.90, 0.96). In addition the Bland-Altman plots indicated that the mean difference between self-reported and direct measurement was relatively small. The mean difference (self-reported minus direct measurements) was, for boys: weight, −2.1 kg; height, −1.6 cm; BMI, −0.44 kg/m^2^ and girls: weight, −1.2 kg; height, −0.9 cm; BMI, −0.3 kg/m^2^. However, 95% limits of agreement were wide which indicated substantial discrepancies between self-reported and direct measurements method at the individual level. Nonetheless, the weighted Kappa statistics demonstrated a substantial agreement between BMI status categorised based on self-reported weight and height and the direct measurements (kappa = 0.76, 95% CI: 0.67, 0.84).

**Conclusion:**

Our results show that the self-reported weight and height were consistent with direct measurements and therefore can be used in assessing the nutritional status of Malaysian school children from the age of 13 to 17 years old in epidemiological studies and for surveillance purposes when direct measurements are not feasible, but not for assessing nutritional status at the individual level.

## Background

Anthropometric indices such as weight and height measurements are used for assessing nutritional status (adequacy of nutrient intake for the maintenance of health and well-being), monitoring physical growth and for early detection of malnutrition among children and adolescents. Though direct measurement of weight and height is preferred in calculating body mass index (BMI), in large epidemiology surveys and surveillance systems [1, 2], self-reported weight and height are commonly used due to time, financial resource and manpower constraints [3]. However, self-reported weight and height are prone to be inaccurate particularly among adolescents, thus its validity needs to be verified as misreporting of weight and height could result in erroneous classification of BMI [4]. There have been conflicting findings about the validity of self-reported data in epidemiological surveys [5–9]. To our knowledge, the validity of self-reported weight and height among Malaysian adolescents has not been studied. Therefore, our study aimed to determine the validity of self-reported weight, height and BMI derived from self-reported weight and height among Malaysian adolescents.

## Methods

Self-reported and measured weight and height data from 663 apparently healthy schoolchildren between 13 and 17 years old in Peninsular Malaysia who are a subgroup of the Malaysian Adolescent Health Risk Behaviour (MyAHRB) survey 2013/2014 participants were analysed. MyAHRB is a cross-sectional, school-based survey on health risk behaviours which was conducted from May to September 2013. This study was registered with the National Medical Research Register and ethical approval was obtained from the Medical Research & Ethics Committee; Ministry of Health Malaysia (Reference number: NMRR-12-1210-12399).

### Sample size calculation

We referred to a published table of sample sizes for inter-rater reliability studies generated based on estimated inter-rater reliability coefficient (kappa statistics), relative error and difference between overall agreement probability and chance-agreement probability [10]. We selected a sample size of 625, which is the minimum sample size assuming that the estimated inter-rater reliability coefficient (kappa statistics) in the sample differs from the “true” value of inter-rater reliability in the population by not more than 20% (relative error) and the difference between overall agreement probability and chance-agreement probability of 0.2.

### Sampling

The study population was all apparently healthy school children in the east coast state of Kelantan. Six schools were randomly selected from 135 secondary schools in the state of Kelantan. For each school, 3 classes were selected from Form 1 to Form 5 (Age-range: 13 to 17 years old). Classes were randomly sampled in most of the schools but some were conveniently selected by the school authorities. All students in the selected classes were included in the study. Students who were absent from class due to extra-curricular activity or other reasons, on the day of the survey, were excluded from the study. A total of 698 school children were recruited in this study. Of the 698 students recruited, 35 were subsequently excluded from the final data analysis due to either not reporting their heights (*n* = 2) or weights (*n* = 3); not reporting both height and weight (*n* = 27) or the reported height or weight values were unreasonably high or low (*n* = 3), resulting in a final sample of 663 students.

### Data collection

Data collection was conducted by trained public health paramedic staff. Informed consent was obtained from the parents prior to the study. Then, students whose parents consented were given a briefing before questionnaire administration. The students were assured that their information will be treated with confidentiality and will only be used for research purposes by the Ministry of Health. Before weight and height measurements were taken, health personnel identified students with pre-existing chronic diseases that affect normal growth such as thalassemia [11] and renal disease [12] and also assessed for apparent physical deformities (e.g. loss of limb). If present, these students would be excluded from having their weight and height measured for this study, but they would still be eligible for the other modules in the parent MyAHRB study. However, no such cases were encountered. Data collection was conducted on a school day during school hours. Eligible students were given a self-administered questionnaire to be filled out in their classroom. In the questionnaire, students were asked to report their most recently measured body weight and height, without informing they were to be measured afterwards. In publicly-funded Malaysian secondary schools, students’ weight and height are measured twice a year, once at the beginning of the school year and again mid-year, as part of the Physical Education subject requirement. In this study, we assume that all students had their last measurements taken at approximately the same time and that their weight and height remained stable till the date of the survey.

### Study instrument

Weight and height measurements were performed by trained public health paramedic staff. Students were asked to remove their shoes and wear only light clothes before their weight and height were measured. Height was measured to the nearest 0.1 cm, from the respondent’s head to toe in an upright standing position with five points of the body touching the wall, using the Seca 206 mechanical measuring tape (Seca GmBH & Co. Kg., Hamburg, Germany). Weight was measured to the nearest 0.1 kg, using Tanita HD-318 digital weighing scales (Tanita Corporation, Tokyo, Japan). Body mass index was calculated as weight (in kilograms) divided by height squared (in metres). Data on brief socio-demographic information were also collected.

### BMI classification

The WHO AnthroPlus software was used to generate a z-score for BMI-for-age for each respondent [13]. The WHO Growth Reference 2007 was then used to classify the respondents as underweight, normal weight, overweight and obese based on the z-scores. The cut-off for underweight was z-score < −2 standard deviations (SD), normal: between ≥ −2SD and ≤ +1SD, overweight: > +1SD or obese: > + 2SD [14]. The cut-off of +1SD is equivalent to the overweight cut-off for adults (> 25.0 kg/m^2^) and the +2 SD cut-off is comparable to the obesity cut-off point for adults (> 30.0 kg/m^2^), at age 19 [15].

### Under-reporting and over-reporting of body weight and height

Under-reporting of weight and height were defined as values of self-reported weight and height exceeding 5% below the directly measured weight and height. Over-reporting was defined as self-reported weight and height exceeding 5% above directly measured weight and height [16].

### Statistical analysis

The Bland-Altman plot is one of the commonly used statistical techniques to assess agreement in the quantitative measurements between two methods [17]. In a Bland-Altman plot, the difference between two measurement is plotted against the average of the two measurements [18]. In this study, we used the Bland-Altman plot to demonstrate the differences between self-reported and directly measured weight, height and BMI against the average of self-reported and directly measured weight, height and BMI. Horizontal lines were drawn at the mean difference, and at the 95% limits of agreement, which are defined as the mean difference ± 1.96 x (Standard deviation of the difference). The mean difference indicates the degree of bias between self-reported and directly measured values. The 95% limits of agreement measures precision of the mean difference; implying how far apart the weight, height and BMI values reported by the respondents and those directly measured were more likely to be for most of the participants [18]. The Intraclass Correlation Coefficient (ICC) is another method to examine agreement between two quantitative measurements [17]. The ICC ranges from 0 (no agreement) to 1 (perfect agreement). Hence, we also computed the ICC for self-reported and directly measured weight and height.

Weighted Kappa statistics was computed to determine the degree of agreement between BMI categorization (underweight, normal weight, overweight and obesity) derived from self-reported values and BMI categorization derived from direct measurements [19]. The degree of agreement was classified into 6 categories according to kappa (κ) value as follows: κ < 0 is less than chance agreement; 0.01 ≤ κ ≤ 0.20 is slight agreement; 0.21 ≤ κ ≤ 0.40 is fair agreement; 0.41 ≤ κ ≤ 0.60 is moderate agreement; 0.61 ≤ κ ≤ 0.80 is substantial agreement; and 0.81 ≤ κ ≤ 1.0 is almost perfect agreement [20]. Sensitivity, specificity, positive predictive value (PPV) and negative predictive value (NPV) of self-reported BMI status were also determined using measured BMI as the reference standard. All statistical analyses were conducted using SPSS ver. 18.0 for Windows (SPSS Inc., Chicago, IL, USA).

## Results

**Fig. 1:**
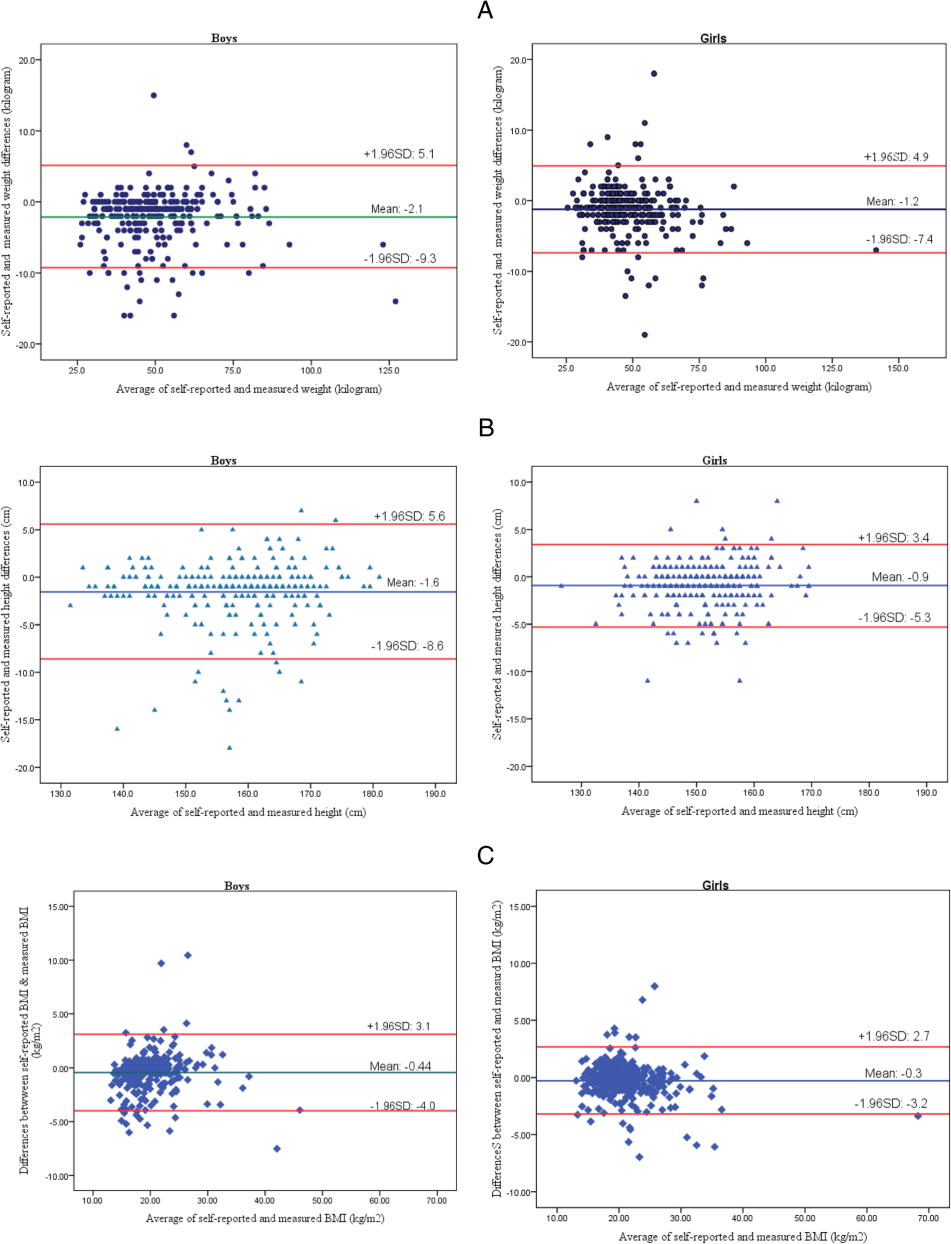
**a** Bland-Altman plot of the differences of self-reported and measured weight compared to the average of self-reported and measured weight for boys (*Left*) and girls (*right*). **b** Bland-Altman plot of the differences of self-reported and measured height compared to the average of self-reported and measured height for boys (*Left*) and girls (*right*). **c** Bland-Altman plot of the differences of self-reported and measured BMI compared to the average of self-reported and measured BMI for boys (*Left*) and girls (*right*)

The selected socio-demographic characteristics of 663 school children are shown in Table 1. Approximately 90% of the school children were Malays and 60% were aged 15 years and below. Only 4.8% (*n* = 32) of the respondents over-reported their weights by an average of 5.1 kg (SD = 3.9) whilst 26.1% (*n* = 173) under-reported their weights by an average of 5.5 kg (SD = 3.4). In terms of height, 0.3% (*n* = 2) of them over-reported their heights by 8 cm (SD = 0) whilst 2.4% (*n* = 16) under-reported their heights by 13.3 cm (SD = 5.4) on average (Table 2). There was excellent intraclass correlation between self-reported and measured weight (*r* = 0.96, 95% CI: 0.93, 0.97) and height (*r* = 0.94, 95% CI: 0.90, 0.96). For boys, the mean difference between self-reported and measured values were −2.1 kg for weight, −1.6 cm for height and −0.4 kg/m2 for BMI. For girls, the corresponding values were −1.2 kg, −0.9 cm and −0.3 kg/m2 (Table 3). The Bland-Altman plot indicated that for boys, the 95% limits of agreement were −9.3 and 5.1 for weight, −8.6 and 5.6 for height and −4.0 and 3.1 for BMI. For girls, the corresponding values were −7.4 and 4.9, −5.3 and 3.4, and −3.2 and 2.7. Larger 95% limits of agreement were observed among boys as compared to girls (Fig. 1a–c).

**Table 1:** Selected socio-demographic characteristics of respondents

	Number	Percent
School locality		
Urban	266	40.1
Rural	397	59.9
Sex		
Male	302	45.6
Female	361	54.4
Ethnicity		
Malay	590	89.0
Chinese	52	7.8
Indian	13	2.0
Others	8	1.2
Age (year)		
13	207	31.2
14	49	7.4
15	147	22.2
16	153	23.1
17	107	16.1

**Table 2:** Under-reporting^a^ and over-reporting^b^ of body weight and height by sex

	Weight (kg)				Height (cm)			
	Under-reporting		Over-reporting		Under-reporting		Over-reporting	
	*n* (%)	Mean (SD)	*n* (%)	Mean (SD)	*n* (%)	Mean (SD)	*n* (%)	Mean (SD)
All (*n* = 663)	173 (26.1)	−5.5 (3.4)	32 (4.8)	5.1 (3.9)	16 (2.4)	−13.3 (5.4)	2 (0.3)	8.0 (0)
Boys (*n* = 302)	96 (31.8)	−5.9 (3.6)	9 (3.0)	5.7 (4.0)	14 (4.6)	−13.6 (5.7)	0 (0)	0 (0)
Girls (*n* = 361)	77 (21.3)	−4.9 (3.2)	23 (6.4)	4.9 (3.9)	2 (0.6)	−11.0 (0)	2 (0.6)	8 (0)

^a^Self-reported weight and height exceeding 5% below directly-measured weight and height

^b^Self-reported weight and height exceeding 5% above directly-measured weight and height

**Table 3:** Means and standard deviations of weight, height and BMI based on self-reported and direct measurement

	Weight (kg)			Height (cm)			BMI (kg/m^2^)		
	Self-reported	Direct measurement	Mean Difference (SD)	Self-reported	Direct measurement	Mean Difference (SD)	Self-reported	Direct measurement	Mean Difference (SD)
	Mean (SD)	Mean (SD)		Mean (SD)	Mean (SD)		Mean (SD)	Mean (SD)	
All (*n* = 663)	47.73 (12.75)	49.34 (13.10)	−1.61 (3.41)	153.83 (8.79)	155.03 (8.67)	−1.21 (2.94)	20.05 (4.63)	20.41 (4.74)	−0.36 (1.61)
Boys (*n* = 302)	48.59 (13.96)	50.65 (14.04)	−2.06 (3.67)	157.17 (9.78)	158.73 (9.47)	−1.56 (3.58)	19.46 (4.49)	19.90 (4.42)	−0.44 (1.77)
Girls (*n* = 361)	47.01 (11.60)	48.24 (12.17)	−1.23 (3.14)	151.03 (6.69)	151.94 (6.49)	−0.91 (2.22)	20.54 (4.69)	20.83 (4.95)	−0.29 (1.47)

*SD* standard deviation

Based on self-report data, prevalence of underweight, normal weight, overweight and obesity were 9.7%, 72.2%, 12.5% and 5.6% respectively. The corresponding rates for directly measured data were 6.0%, 75.1%, 12.2% and 6.6%, respectively. Weighted Kappa statistics analysis showed that, overall, there was a substantial agreement between the BMI classifications derived from self-reported and directly measured data (*ҡ* = 0.76, 95% confidence interval: 0.67, 0.84) (Table 4). Between the sexes, there was a higher agreement among girls compared to boys (Tables 4). The sensitivity and specificity of self-reported BMI for identifying overweight adolescents were 75.3% and 96.2%, respectively; and for obesity 75.0% and 99.4%, respectively (Table 5). There was no marked difference in sensitivity and specificity of self-reported BMI for identifying overweight and obese adolescents by sex. The PPV of BMI-for-age for all respondents for overweight and obesity were 73.5% and 89.2% respectively. The corresponding negative predictive values were 96.6% and 98.2% (Table 5).

**Table 4:** Prevalence of underweight, normal weight, overweight and obesity^a^ based on self-report and direct measurements

	Self-reported *n* (%)				Directly-measured *n* (%)				_Ҡ_^b^ value	95% CI
	Underweight	Normal weight	Overweight	Obesity	Underweight	Normal weight	Overweight	Obesity		
All	64 (9.7)	479 (72.2)	83 (12.5)	37 (5.6)	40 (6.0)	498 (75.1)	81 (12.2)	44 (6.1)	0.76	0.67, 0.84
Boys	42 (13.9)	208 (68.9)	34 (11.3)	18 (6.0)	22 (7.3)	227 (75.2)	33 (10.9)	20 (6.6)	0.72	0.59, 0.85
Girls	22 (6.1)	271 (75.1)	49 (13.6)	19 (5.3)	18 (5.0)	271 (75.1)	48 (13.3)	24 (6.6)	0.79	0.67, 0.90

^a^WHO Growth Reference, 5–19 years (2007)

^b^Weighted kappa statistic (Absolute error weighting method)

**Table 5:** Sensitivity, specificity, positive predictive value and negative predictive value of self-reported BMI classifications^a^ by sex

	All				Boys				Girls			
Self-reported BMI	Sn (%)	Sp (%)	PPV (%)	NPV (%)	Sn (%)	Sp (%)	PPV (%)	NPV (%)	Sn (%)	Sp (%)	PPV (%)	NPV (%)
Underweight	77.5	94.7	48.4	98.5	81.8	91.4	42.9	98.5	72.2	97.4	59.1	98.5
Normal weight	90.6	83.0	94.2	74.5	86.3	84.0	94.2	67.0	94.1	82.2	94.1	82.2
Overweight	75.3	96.2	73.5	96.6	72.7	96.3	70.6	96.6	77.1	96.2	75.5	96.5
Obesity	75.0	99.4	89.2	98.2	80.0	99.3	88.9	98.6	70.8	99.4	89.5	98.0

*Sn* sensitivity, *Sp* specificity, *PPV* positive predictive value, *NPV* Negative predictive value

^a^WHO Growth Reference, 5–19 years (2007)

## Discussion

Generally, our results suggest that Malaysian adolescents tend to under-report their weight as this study showed that approximately a quarter of them (26.1%) under-reported their weight, while only less than 5% over-reported their weight and misreported their height. Our findings were in line with previous studies in Korea [21], Greece [22], Portugal [23], Germany [24] and the United States [6, 25]. The consequences of under-reporting weight are overestimation of the prevalence rate of underweight, and underestimation of obesity. The magnitude of discrepancy between self-reported and measured weight and height as well as the corresponding BMI could be related to age, sex, ethnicity, socio-economic variation (parental socioeconomic status), BMI status, self-perception of body weight and desire to control one’s weight, pubertal status as well as health related lifestyle factors (physical activity, smoking, sedentary behaviour, vegetable and fruit consumption). Previous studies among adolescents in US [26, 27], and Sweden [8] showed that being female and being overweight were significantly associated with under-reporting of BMI. Other factors such as age, ethnicity, health-related behaviours and pubertal status were not significantly associated with biased reporting of BMI. Among Greek [22] and Chinese adolescents [9], accuracy of self-reports are reportedly influenced by age, whereby older adolescents are more likely to under-report their weight compared to younger adolescents. In a US study, this trend was reported only among female adolescents [27]. On the contrary, in a study among Estonian adolescents, the trend was in the opposite direction, with more accurate self-reporting by older adolescents [28]. In addition, Jayawardene and colleagues [25] analysed data from the 2010 US National Youth Physical Activity and Nutrition Survey which showed that US adolescents who were obese or were trying to lose weight tended to under-report their weight, and consequently underestimated their BMI. In another study, by Brettschneider et al. [24], misperception of one’s body weight and having parents who are both overweight could lead to misclassification of BMI status among German adolescents, but other factors such as socio-economic status, sexual maturation and eating disorder were not significantly associated with bias in self-reported weight and height.

The validity of self-reported BMI may be assessed by its sensitivity, specificity and predictive values in classifying the respondents into overweight and obese, using BMI derived from direct measurements of weight and height as reference. A high sensitivity means that fewer overweight or obese subjects would be misclassified as non-overweight or non-obese when using BMI derived from self-reported weight and height. In other words, those truly overweight or obese respondents would be correctly identified as such when using self-reported data. On the other hand, a high specificity means that fewer non-overweight or non-obese subjects would be misclassified as overweight or obese and more truly non-overweight or non-obese respondents would be correctly identified as such when using self-reported data [3]. Generally, our study showed that the estimated overall sensitivity (75%) and specificity (>95%) of overweight and obesity based on self-reported data were fairly good. The present findings were comparable to findings reported by Sherry et al. [26] in which a review of previous studies among the US population demonstrated that the sensitivity of BMI in identifying overweight adolescents based on self-reported data ranged from 55% to 76%. Nonetheless, studies conducted by Zhou et al. [9] among Chinese adolescents and by Ekström et al. [8] among Swedish adolescents showed a lower sensitivity of self-reported overweight and obesity. Sensitivity of self-reported overweight among Chinese adolescents was 56.1% [9] and among Swedish adolescents, 60.2% and 46% for overweight and obesity, respectively [8]. The PPV of BMI-for-age indicates that approximately 50% of underweight respondents based on self-report were truly underweight based on direct measurement, and approximately 75% and 90% of overweight and obese respondents were truly overweight and obese, respectively. The NPV, on the other hand, showed that almost all respondents classified as non-overweight or obese based on self-report were indeed not overweight or obese by direct measurement.

It was noted that the sensitivity of self-reported BMI in identifying obesity was higher among boys than girls in the present study. Self-reported BMI misclassified 27.3% and 20% of overweight and obese boys as non-overweight and non-obese, respectively. Among girls, 22.9% of overweight and 29.2% of obese girls were misclassified. These findings were consistent with previous studies among the adolescents in Korea (sensitivity of self-reported obesity were 71.4–74.1% among boys and 57.1% -60.0% among girls) [21] and Germany (75.8% among boys and 73.7% among girls) [24]. Nonetheless, it should be noted that different countries apply different country-specific cut-offs in classifying overweight and obesity, therefore, direct comparisons of sensitivity of self-reported data in BMI classification between countries may not be appropriate and should be interpreted with caution. Generally, the sensitivity of self-reported BMI in classifying overweight and obesity were frequently higher among boys than girls. The reason for this could be the difference between measured and self-reported weight tend to be bigger among girls who underreported their weight than among their male counterparts [8, 25, 26, 28]. Besides, thinness among adolescent girls is socially desirable. Widespread overemphasis on equating thinness with beauty and success in the mass media, parental and peer pressure to be thin, are possible explanations for under-reporting of body weight [29, 30]. Consequently, under-reporting of BMI was more common among girls, and the magnitude of under-reporting was also higher among girls compared to boys.

In the present study, weighted kappa statistics indicated that there was a substantial agreement (κ =0.76) in the BMI classification based on self-reported and directly measured data. In spite of using country-specific cut-offs points in defining BMI status and differences in number of BMI classifications, comparable results were reported in previous studies across different countries such as China (κ = 0.75) [9], Korea (κ = 0.79) [21] and Belgium (κ = 0.67) [5]. It could be deduced that the BMI status derived from self-reported weight and height could be accepted as an alternative measure for the assessment and surveillance of nutritional status among Malaysian adolescents, when direct measurement of weight and height is impractical [6]. However, the prevalence of overweight or obesity based on self-reported data should be interpreted with caution as it could be an underestimate of the true prevalence. Also, cross-comparisons of the prevalence of overweight and obesity derived from self-reported data with other groups or communities should not be performed since the factors related to the biases of reporting in different groups or communities may be significantly different [3].

The present study showed that the intraclass correlation coefficients between self-reported and directly measured weight, height and BMI were high and this indicated a high agreement between self-reported and directly-measured data. Furthermore, the Bland-Altman plots of the differences between self-reported and measured weight, height and BMI against respective means were satisfactory. However, the 95% limits of agreement were notably large for both boys and girls which indicated merely fair agreement between self-reported and direct measured data. Similar findings were also reported in studies among Portuguese [23] and Chinese adolescents [9]. The large 95% limits of agreement are attributed to high variability of self-reported data at the individual level as the plots showed the shift of few data points beyond the limits [31]. Therefore, self-reported data should not be applied in assessing and monitoring nutritional status at the individual level especially in clinical settings as a basis for diagnosis of malnutrition and recommendation of nutritional intervention [3].

The study sample comprised of adolescents who attended secondary school during the schooling period and therefore it did not represent all adolescents in the population. Furthermore, not all school children in the selected classes participated in the study as some were involved in curricular activities outside the classroom or were absent from school during data collection. Besides, self-reported weight and height relies on response capability which could be influenced by recent weight- and height-measuring history as well as the recall ability of the individual adolescent [32].

## Conclusion

Self-reported weight and height were proven to have high correlation with direct measurements, subsequently self-report derived BMI may be used in assessing the nutritional status of Malaysian school children in epidemiological studies and for surveillance purposes when direct measurements are not feasible. However, self-reported weight and height are not recommended for assessing nutritional status at the individual level.

## Abbreviations

95% CI: 95% confidence interval
BMI: Body mass index
ICC: Intraclass correlation
MyAHRB: Malaysian adolescent health risk behaviour
NPV: Negative predictive value
PPV: Positive predictive value
SD: Standard deviation
